# Bovine Genome Database: new annotation tools for a new reference genome

**DOI:** 10.1093/nar/gkz944

**Published:** 2019-10-24

**Authors:** Md Shamimuzzaman, Justin J Le Tourneau, Deepak R Unni, Colin M Diesh, Deborah A Triant, Amy T Walsh, Aditi Tayal, Gavin C Conant, Darren E Hagen, Christine G Elsik

**Affiliations:** 1 Division of Animal Sciences, University of Missouri, Columbia, MO 65211, USA; 2 Division of Environmental Genomics and Systems Biology, Lawrence Berkeley National Laboratory, Berkeley, CA 94608, USA; 3 Department of Bioengineering, University of California, Berkeley, CA 94720, USA; 4 Bioinformatics Research Center, North Carolina State University, Raleigh, NC 27695, USA; 5 Department of Biological Sciences, North Carolina State University, Raleigh, NC 27695, USA; 6 Program in Genetics, North Carolina State University, Raleigh, NC 27695, USA; 7 Department of Animal and Food Sciences, Oklahoma State University, Stillwater, OK 74078, USA; 8 Division of Plant Sciences, University of Missouri, Columbia, MO 65211, USA; 9 MU Institute for Data Science and Informatics, University of Missouri, Columbia, MO 65211, USA

## Abstract

The Bovine Genome Database (BGD) (http://bovinegenome.org) has been the key community bovine genomics database for more than a decade. To accommodate the increasing amount and complexity of bovine genomics data, BGD continues to advance its practices in data acquisition, curation, integration and efficient data retrieval. BGD provides tools for genome browsing (JBrowse), genome annotation (Apollo), data mining (BovineMine) and sequence database searching (BLAST). To augment the BGD genome annotation capabilities, we have developed a new Apollo plug-in, called the Locus-Specific Alternate Assembly (LSAA) tool, which enables users to identify and report potential genome assembly errors and structural variants. BGD now hosts both the newest bovine reference genome assembly, ARS-UCD1.2, as well as the previous reference genome, UMD3.1.1, with cross-genome navigation and queries supported in JBrowse and BovineMine, respectively. Other notable enhancements to BovineMine include the incorporation of genomes and gene annotation datasets for non-bovine ruminant species (goat and sheep), support for multiple assemblies per organism in the Regions Search tool, integration of additional ontologies and development of many new template queries. To better serve the research community, we continue to focus on improving existing tools, developing new tools, adding new datasets and encouraging researchers to use these resources.

## INTRODUCTION

The availability of multiple livestock reference genome sequences has accelerated genomics based livestock research and breeding programs (1). The Bovine Genome Database (BGD) (2) provides easy access to the bovine genome assemblies and annotations via graphical genome browsers (JBrowse) (3), annotation tools (Apollo) (4), sequence search (BLAST) (5,6) and data mining (BovineMine) (2,7). Genome browsers at BGD provide different genomic features such as protein-coding and non-coding genes from RefSeq (8) and Ensembl (9), single nucleotide polymorphisms (SNP) from Ensembl Variation (9), quantitative trait loci (QTL) from AnimalQTLdb (10), as well as expression tracks computed using publicly available RNA-seq data (NCBI Sequence Read Archive (11)). BovineMine integrates these data with other external sources, including protein annotation (UniProt (12)), protein families and domains (InterPro (13)), orthologs and paralogs (EnsemblCompara (14), OrthoDB (15), TreeFam (16)), pathways (KEGG (17), Reactome (18)), interactions (BioGRID (19), IntAct (20)), Gene Ontology annotations (UniProt GOA (21)), SNP alias ids (SNPChiMp (22)) and publications (PubMed (11)).

BGD was first published in *Nucleic Acids Research* in 2011 (23), with the initial goal of supporting the Bovine Genome Sequencing and Analysis Consortium (BGSAC) (24) by providing web-based tools for annotation of the bovine genome. An update in the NAR database issue five years later (2) reported new genome browsing and annotation tools using JBrowse (3) and Apollo (25) for two *Bos taurus* genome assemblies, the reference genome assembly (UMD3.1.1) (26) and the alternate genome assembly (Btau_4.6.1) (24), as well as a data mining tool called BovineMine, based on the InterMine data warehousing platform (27). Since then we have been making continuous improvements to our existing tools, adding new tools and data and updating existing datasets.

## LSAA: NEW TOOL FOR ANNOTATION OF GENOME ASSEMBLIES

**Figure 1.**
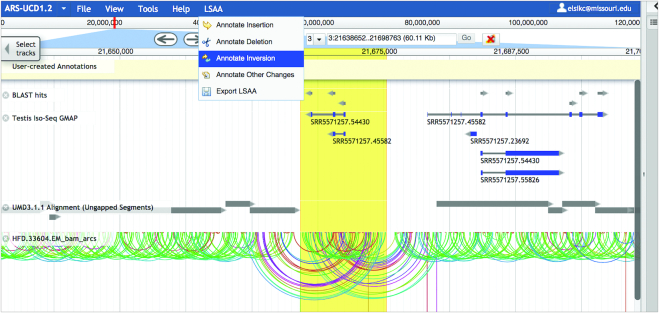
Apollo browser showing the LSAA pulldown menu and evidence for a genome assembly inversion. The *BLAST hits* track shows that segments of a single cDNA align to the genome in opposite directions and the *Testis Iso-Seq GMAP* track shows that some full-length Iso-Seq transcripts have partial alignments in opposite directions. *The UMD3.1.1 Alignment (Ungapped Segments)* track reveals a region without an alternate assembly alignment. In the *HFD_33604_EM_bam_arcs* track, connections between aligned mate pairs of a genomic read library show unusually large overlapping arcs that suggest an assembly inversion. The region of the suspected assembly inversion is highlighted using the highlight tool to the right of the navigation box, and the LSAA pulldown menu can be used to annotate the region as an inversion.

**Figure 2.**
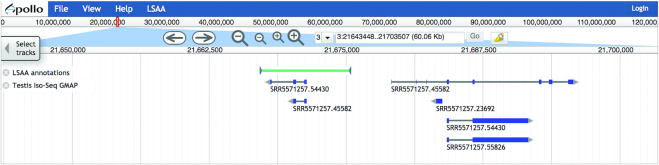
LSAA track in JBrowse. Once a tentative assembly inversion has been annotated, it is viewable as a track in JBrowse. The inversion is depicted as a green bar with inverted arrowheads.

Although sequencing technologies and genome assembly algorithms have improved over the years, reference genome assemblies still contain errors that can impact gene annotation (28). We have created the Locus-Specific Alternate Assembly (LSAA) tool, developed as a plugin for Apollo, which enables users to manually correct local assembly errors or provide data about genomic structural variants, such as breed specific variants, in the genome browser. The user-submitted LSAA annotations provide the locations and alternative sequences to correct assembly errors or describe variants. We added database objects for alternative loci to the Apollo 2.0 codebase, which utilizes the Chado schema (29), allowing LSAA to be saved in the Chado database. By leveraging Apollo, our tool allows users to curate LSAA while they annotate genes. LSAA user features include (i) a pulldown menu that opens a box in which users can enter coordinates and provide sequences and descriptions to create LSAA annotations (Figure 1); (ii) a Paired Arc viewer to assist the visualization of mate pair BAM files, with arcs that connect the read pairs and can highlight possible assembly issues (Figure 1); (iii) a combined LSAA report and export tool that enables searching and filtering; (iv) a LSAA track with custom set of glyphs that represent different assembly changes (Figure 2).

An example of the application of the LSAA tool is provided in the Supplementary Data. Researchers often come across assembly errors in the process of manual gene annotation. As an example, an assembly inversion can be indicated when different segments of a single cDNA sequence align on opposite strands of the assembly or when the distance between mate pair reads in a genomic mate pair library align at larger or shorter distances from each other than expected (Figure 1). Supplementary Figure S1 shows a BLAST search with a cDNA sequence, resulting in an unexpected alignment pattern seen in JBrowse, followed by further investigation with additional JBrowse tracks. Supplementary Figures S2–S4 show the use of the LSAA tool to annotate an inversion, and Supplementary Figures S5–S6 show how to export and view submitted LSAA.

The LSAA tool provides visualizations to help users pinpoint assembly issues. Integration with a BLAST search tool enables visualizing BLAST high-scoring segment pairs (HSPs) on the browser (Supplementary Figure S1). A Paired Arc track displays genomic read alignments as arcs between read pairs, highlighting discordant read pairs as unusually large arcs (Figure 1 and Supplementary Figure S2). While the BLAST and Paired Arc visualizations are available prior to logging in to Apollo, users must be logged in to Apollo (described below) in order to submit and export LSAA annotations (Supplementary Figure S3). The menu for LSAA creation allows for the submission of locations for insertions, deletions, inversions and other changes, such as substitutions, as well as the entry of associated information, such as breed, individual, description and alternative sequence (Supplementary Figure S4). Once LSAA annotations are submitted, they are maintained, viewable, searchable, and downloadable but the reference assembly is not permanently changed. Results can be instantaneously shared with other bovine researchers. A filterable table of submitted LSAA annotations provides functions for navigating to LSAA regions in the browser; exporting LSAA FASTA sequences and schematic representations in JSON; and deleting LSAA (Supplementary Figure S5). LSAA annotations can be viewed on the browser by selecting the LSAA annotations track (Figure 2). Right clicking a LSAA feature opens a panel showing annotation details (Supplementary Figure S6). Code for the LSAA plugin for Apollo is available on GitHub (https://github.com/elsiklab/Apollo-LSAA) for use by other genome database providers.

## JBROWSE/APOLLO: NEW AND OLD ASSEMBLIES

Previously, BGD had genome browsers for the UMD3.1.1 bovine reference genome assembly and an alternate genome assembly, Btau_4.6.1. A new bovine reference genome, ARS-UCD1.2, with notably improved contiguity, was recently released (30). BGD currently provides JBrowse for both the new ARS-UCD1.2 reference and the former UMD3.1.1 reference genome, which can be selected from the JBrowse pulldown menu in the BGD navigation bar. The ‘Alternate Assembly Alignment’ tracks available in each browser show the results of whole genome alignment and chaining using BLAT and associated tools (31). Each browser includes tracks representing either the ungapped genome segments or the result of chaining the ungapped segments together. Clicking an ungapped segment opens a new JBrowse window of the alternate assembly, zoomed into the region of the aligned segment. Using this track in combination with viewing gene prediction tracks enables users to navigate across assemblies to examine genes in corresponding regions. The availability of browsers for both the UMD3.1.1 and ARS-UCD1.2 bovine reference genomes with large numbers of transcript and genomic data tracks will be valuable as researchers transition from the older to the newer bovine reference genome assembly.

Apollo annotation tools for the ARS-UCD1.2 genome assembly are available after logging in using a box in the upper right corner of the JBrowse window. The Apollo registration page, accessible from the Apollo pulldown menu on the BGD navigation bar, provides instructions for non-registered users who wish only to view existing annotations. Users must register using a valid email address to access all Apollo functions. With a user account, researchers can view and export all existing user annotations, manually edit genes and annotate new genes. An additional Apollo instance, the Bovine Apollo Demo, also available using the Apollo pulldown menu on the BGD navigation bar, provides full Apollo functionality with a guest login, but is intended for annotation practice, not for maintaining user submitted annotations.

## BGD BLAST: IMPROVED INTEGRATION WITH APOLLO

The BGD BLAST search tool, which is based on the Sequenceserver platform (6), is accessible from the BGD navigation bar. BLAST hits are linked to JBrowse based on match coordinates when a genome assembly is used as the search database (Supplementary Figure S1). Previously, the blast hits could be viewed only as highlighted regions in the genome. Now alignments of individual BLAST HSPs and their directionality can be viewed and used as evidence in a gene or LSAA annotation. Furthermore, when logged into Apollo, the HSP features can be dragged to the user annotation area to initiate gene annotations.

## GENE EXPRESSION DATA: UPDATED AND NEW

In the past, BGD had gene expression tracks in JBrowse and computed gene expression levels in BovineMine for UMD3.1.1, based on single-end RNA-seq data for 95 tissues (NCBI BioProject PRJNA263600). We have re-processed the single-end RNA-seq data with the new reference genome, ARS-UCD1.2, and have added 40 paired-end RNA-seq datasets (NCBI BioProjects PRJNA379574 and PRJNA294306). Our methods for computing JBrowse tracks and BovineMine gene expression levels were updated from the previous TopHat/Cufflinks pipeline (32,33), to the improved Hisat2/StringTie pipeline (34,35). In addition, 23 long-read transcriptome datasets (Iso-Seq) were added to JBrowse/Apollo (NCBI BioProjects PRJNA386670 and PRJNA434299). Multiple Iso-Seq and RNA-seq visualizations are available for each dataset.

## BOVINEMINE: ADDED FEATURES AND UPDATES

BovineMine has undergone five new releases since the previous BGD update publication (2). The current release is BovineMine v1.6. Each new release incorporated new and updated data, with better integration, improved queries and refined user interfaces. Detailed examples for the application of BovineMine were published (7) using the BovineMine v1.3 release, which is still available on the BGD homepage. BovineMine enhancements are described below.

### Multiple ruminant species and genome assemblies

BovineMine now hosts multiple genome assemblies of *Bos taurus* (UMD3.1.1 and ARS-UCD1.2), as well as genome assemblies of other ruminants: *Ovis aries* (Oar_v3.1 and Oar_v4.0) (36,37) and *Capra hircus* (ARS1) (38). Because the transition to a new genome can be difficult for researchers in the midst of ongoing projects, BovineMine has template queries that support conversion between gene sets and assemblies. Users have the choice of continuing research on the older assembly or using the new assembly. Template queries for converting between genomes and gene sets combined with the previously described List Tool (2) make it possible to perform meta-analysis of published research using both the old and new genome assemblies. The ability to convert between assemblies is especially important for the bovine research community, for which there is 10 years of published research on the older assembly.

With the availability of multiple assemblies per organism in BovineMine, we needed to modify the Regions Search tool, which allows users to perform a chromosome and coordinate based search for genomic features. In the past, the Regions Search tool allowed only one assembly per organism and users could select specific organism from a dropdown menu. The Regions Search tool now has an additional pulldown menu to select the assembly.

### New ortholog and metabolic datasets

BovineMine contains a new ortholog dataset called ORIS (ORthology Inference using Synteny), which includes bovine, human, mouse, sheep and pig genes (39). In addition, we incorporated a new metabolic reconstruction that was developed from the ORIS ortholog set based on the RECON human metabolic network reconstruction (40) using previously reported methods (41). BovineMine enrichment widgets allow testing gene lists for enrichment of metabolic subsystems and reactions.

### New ontologies

Previously the only ontologies supported by BovineMine were Sequence Ontology (42), Gene Ontology (43,44) and Mouse Anatomy Ontology (45). We have added several new ontologies, including Vertebrate Trait Ontology (46), Evidence Code Ontology (47), Uberon Anatomy Ontology (48), Protein–Protein Interaction and Molecular Interaction Ontology (49), Livestock Breed Ontology (https://www.animalgenome.org/bioinfo/projects/lbo/) and Livestock Product Trait Ontology (https://www.animalgenome.org/bioinfo/projects/lpt/).

### Other BovineMine enhancements

In addition to the major BovineMine enhancements described above, several minor enhancements were implemented. There are multiple datasets where improvements were made in the data integration step. For example, QTL in BovineMine are now integrated with flanking SNP and relevant ontologies; expression metadata in BovineMine are now linked with relevant anatomy and tissue ontologies; orthology data in BovineMine can now be filtered by last common ancestor; and many new template queries have been added.

## MISCELLANEOUS BGD IMPROVEMENTS

We have made additional improvements and updates to the BGD website. The BGD website itself and all underlying applications have been moved to a new server, resulting in notable improvements in the responsiveness of BovineMine and JBrowse, and the BGD website has been completely revamped. Several software applications, including Apollo, InterMine and the Drupal content management system have been updated. The search tools previously accessible via ‘Search and Annotation Tools’ on the main BGD navigation bar, and designed to help annotators select tracks or chromosomal locations, have now been incorporated into BovineMine as template queries.

## ACCESSING BGD DATA AND TOOLS

BGD tools and data are freely available at http://bovinegenome.org, but some tools require login for full functionality. All data hosted at BGD, other than user submitted gene annotations, are accessible without logging in using JBrowse, BovineMine, Sequenceserver BLAST and the data download page. As described above, free Apollo registration is required for submitting or editing annotations using Apollo and the LSAA Tool. The Apollo registration page also provides guest login instructions for those who wish to view or export user annotations without editing. A separate Apollo instance, the Bovine Apollo Demo, is available with guest login for those who wish to practice annotating genes, but the practice annotations are not permanently maintained at BGD. BovineMine is freely available without logging in, but registering for a BovineMine account, by clicking ‘Log in’ on the BovineMine home page, provides several advantages. When users are logged in to BovineMine, query histories are automatically saved, and users can save queries, query templates and lists for later sessions and for sharing with other users. In summary, the only aspect of BGD that requires user registration is the ability to submit and modify annotations using the main (non-demo) bovine Apollo instance, whereas user registration for BovineMine is advantageous but not required.

## CITING BGD

Cite this article for the use of BGD tools such as BovineMine, JBrowse, Apollo, BLAST and code available on GitHub.

## CONCLUSIONS

To better serve the bovine research community, we have incorporated new and updated data, improved existing tools and developed new tools. In particular, the new LSAA Tool will allow the research community to curate both assembly errors and genomic structural variants among breeds and individuals, with submitted LSAA annotations immediately available for viewing and downloading by others. The LSAA annotations will both alert researchers of potential issues near genes of interest and provide valuable input to future bovine genome assembly upgrades. To support transition to the new reference genome, both BovineMine and JBrowse facilitate comparing old and new genomes and gene sets. Furthermore, BovineMine is the only resource that facilitates meta-analysis of published research results using the old and new bovine genome assemblies.

We will continue to enhance the Bovine Genome Database by adding new datasets and new tools to support bovine research community. BovineMine will be updated and released on an annual basis. Source code modifications will continue to be available on GitHub (https://github.com/elsiklab/). Most importantly, we encourage dataset suggestions and feedback from the bovine research community to guide future development.

## Supplementary Material

gkz944_Supplemental_File
