# Supplementary material for: Bovine Genome Database: new annotation tools for a new reference genome
Source: Nucleic Acids Res. 2019 Oct 24;48(D1):D676–81. doi: 10.1093/nar/gkz944 (PMC7145693; doi:10.1093/nar/gkz944)
Supplement: gkz944_Supplemental_File [file gkz944_supplemental_file.pdf]

Supplementary Data

Bovine Genome Database: New Annotation Tools for a New Reference Genome

Md Shamimuzzaman, Justin J. Le Tourneau, Deepak R. Unni, Colin M. Diesh, Deborah A. Triant, Amy T. Walsh, Aditi Tayal, Gavin C. Conant, Darren E. Hagen, Christine G. Elsik

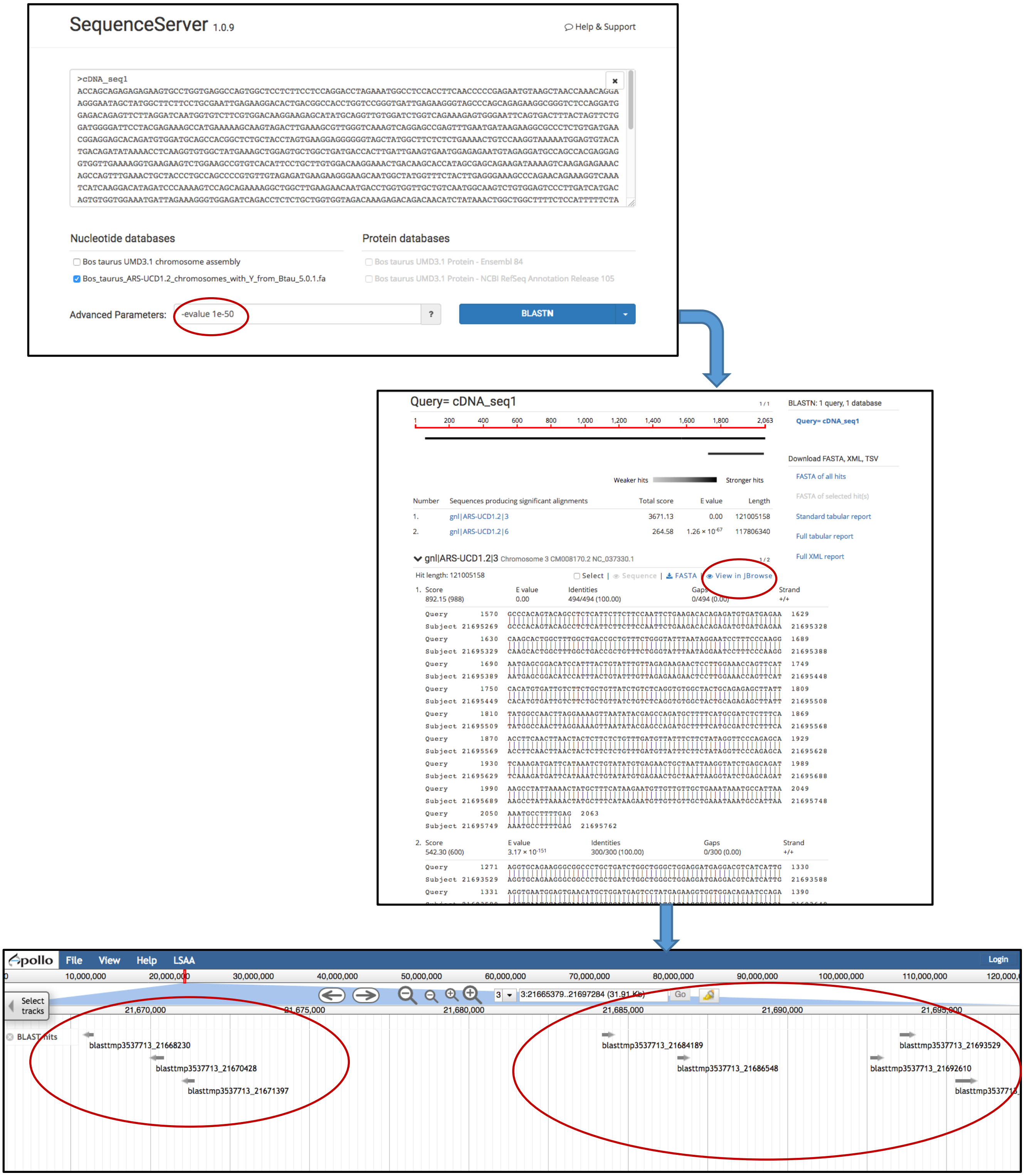

Figure S1. BLAST is used to align a bovine cDNA sequence to the ARS-UCD1.2 genome assembly. The evalue parameter is set to a stringent value of 1e-50 to avoid spurious matches, since the cDNA sequence should be nearly identical to the genome. Clicking "View in JBrowse" above the alignment in the BLAST result page opens JBrowse and shows the BLAST high scoring pairs (HSPs) with arrows indicating direction of each alignment. HSPs aligned in opposite directions may indicate an inversion in the assembly.

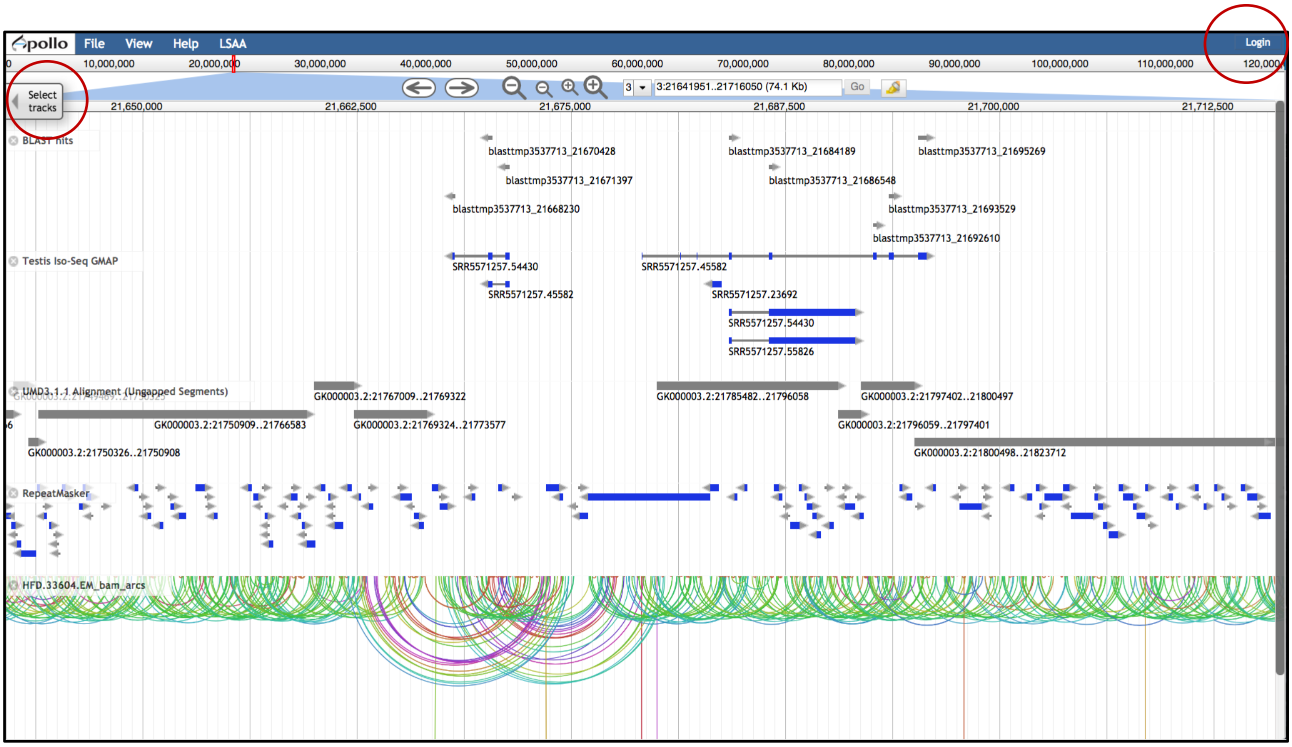

Figure S2. The Select Tracks tab in the upper left of the JBrowse view has been used to access the Faceted Track Selector to select additional tracks to investigate a potential assembly inversion. The *Testis Iso-Seq GMAP* track shows that some full-length Iso-Seq transcripts have partial alignments in opposite directions. *The UMD3.1.1 Alignment (Ungapped Segments)* track reveals a region without an alternate assembly alignment. The *RepeatMasker* track shows a large repeat near the region of interest (Smit, AFA, Hubley, R & Green, P. RepeatMasker Open-4.0.2013-2015 http://www.repeatmasker.org) . The *HFD_33604_EM_bam_arcs* track is a Paired Arc view showing connections between mate pairs in a genomic read library from Dominette, the reference individual. Unusually large overlapping arcs suggest an assembly inversion which causes increased distance between mate pair reads. With this combined evidence, we would like to annotate a LSAA. The first step in annotation is to login to Apollo using the Login button in the upper right corner.

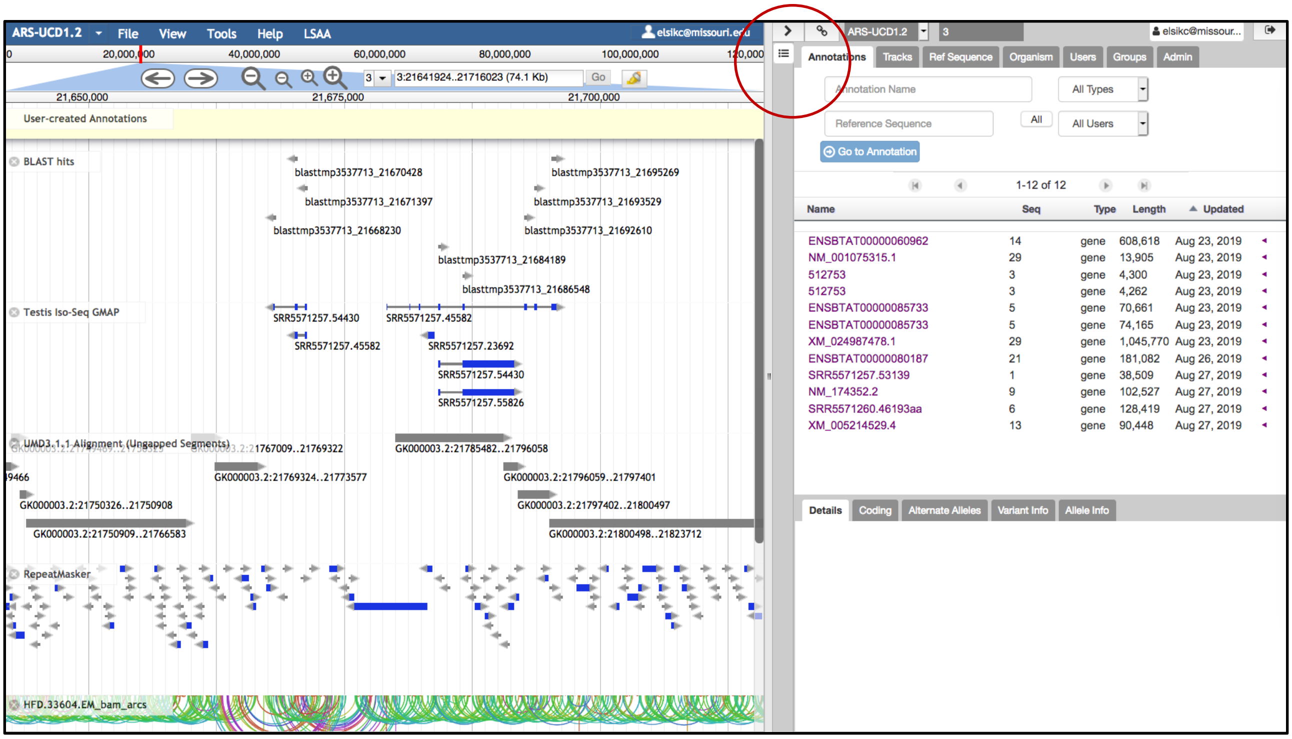

Figure S3. This shows the view once logged into JBrowse. Most users will see only the Annotations, Tracks and Ref Sequence tabs in the Information Panel on the right, while admins will see all the tabs. The Information Panel can be hidden to increase the browser viewing area using the small ">" in the upper left of the Information Panel. Clicking the list icon under the ">" brings back the "Select Tracks" option that is present in the upper left of the JBrowse view in the previous figures.

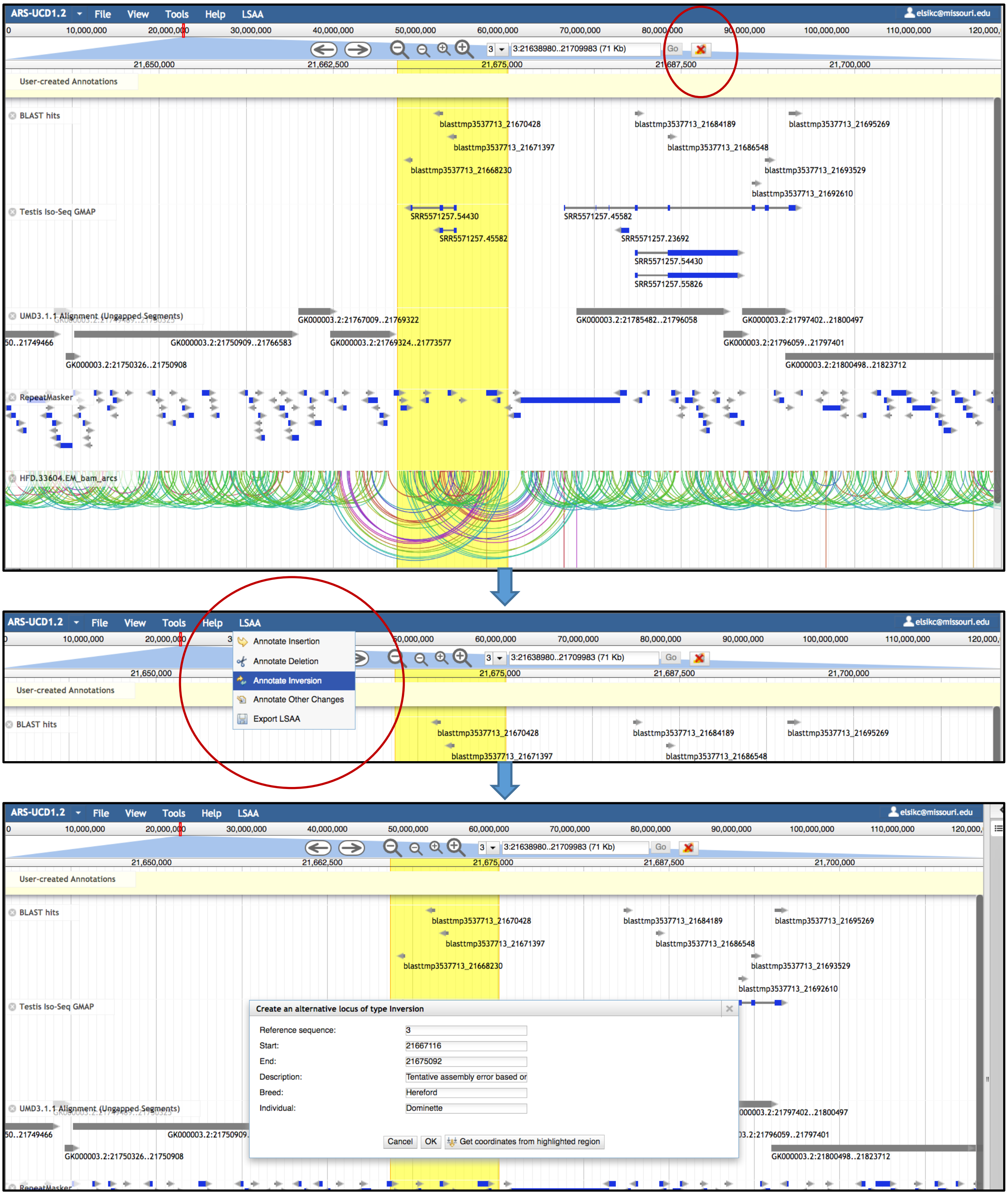

Figure S4. Clicking an icon to the right of the JBrowse coordinate search box allows highlighting the suspected assembly inversion region. The LSAA pulldown menu includes an option to annotate an inversion. Selecting that option provides a small web form in which we can select "Get coordinates from highlighted region" to enter the coordinates of the tentative inversion. Additional information can by typed into the Description box, and the breed and individual can be entered. The annotation is submitted by clicking "OK".

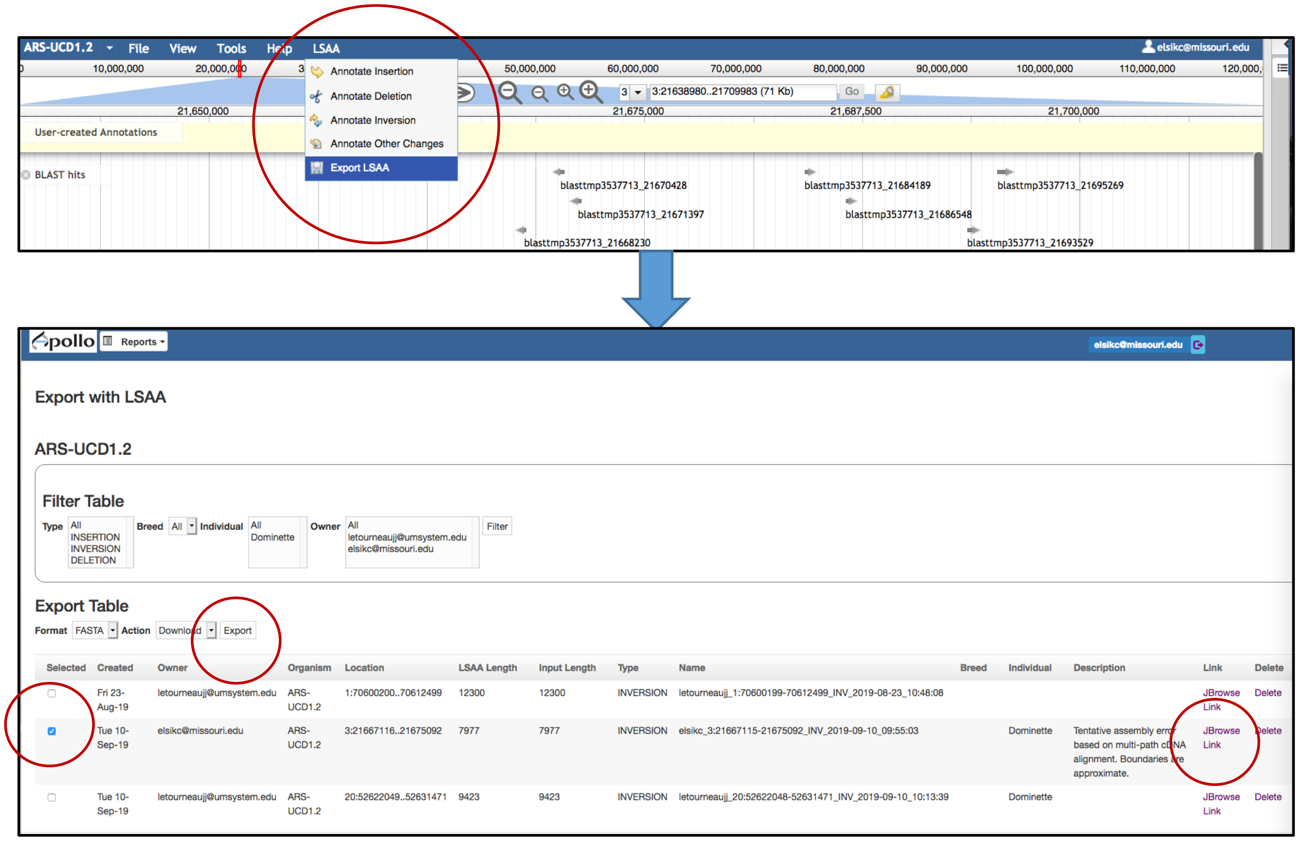

Figure S5. The LSAA pulldown menu can be used to both view and export LSAA, by selecting Export LSAA in the pulldown menu. The resulting report page contains a table of all submitted LSAA. The table can be filtered by type of assembly change, breed, individual and user. Clicking "JBrowse Link" next to an individual LSAA annotation opens a new JBrowse window in the region of the selected annotation. Boxes at the left of each row allow selection of LSAA to incorporate into a new chromosome sequence for download. Multiple LSAA can be selected as long as they do not overlap with each other. The "Export" button allows exporting a new chromosome sequence in FASTA format, or LSAA information in JSON format.

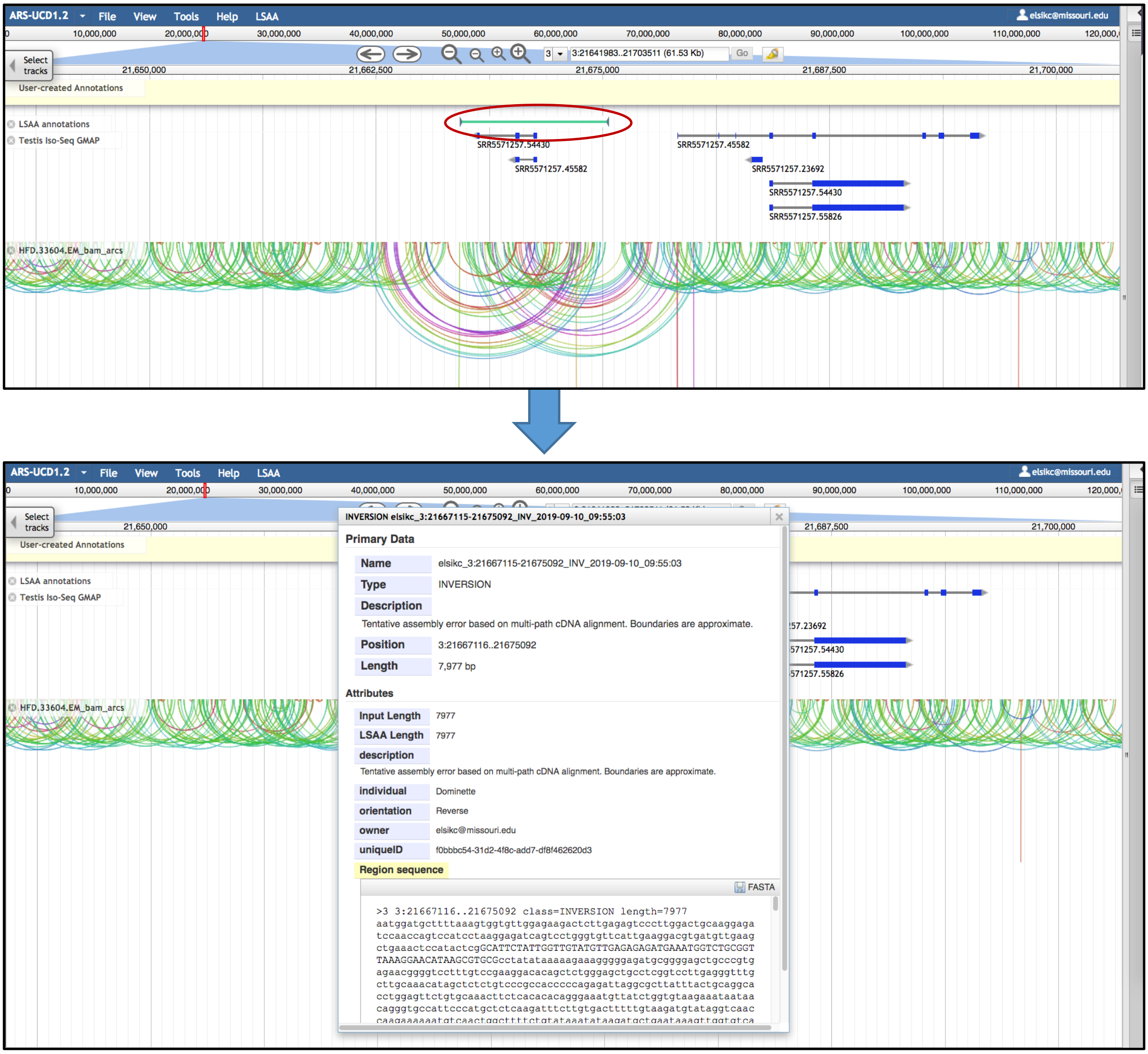

Figure S6. User submitted LSAA can be viewed on the browser by selecting the LSAA annotations track. Each type of change has a specific glyph: a green bar surrounded by inverted gray arrowheads for inversions, a red bar surrounded by inverted gray arrowheads for deletions, and inverted gray arrowheads without an intervening bar for insertions. Right or control-clicking the glyph opens a panel showing annotation details.
